# Remote Evaluation of Rotational Velocity Using a Quadrant Photo-Detector and a DSC Algorithm

**DOI:** 10.3390/s16050587

**Published:** 2016-04-25

**Authors:** Xiangkai Zeng, Zhixiong Zhu, Yang Chen

**Affiliations:** School of Optoelectronic Information, Chongqing University of Technology, Chongqing 400054, China; 13677610413@139.com (Z.Z.); chenyangbisheng@163.com (Y.C.)

**Keywords:** remote evaluation, rotational velocity, differential subtraction correlation, quadrant photodetector, temporal-delay number

## Abstract

This paper presents an approach to remotely evaluate the rotational velocity of a measured object by using a quadrant photo-detector and a differential subtraction correlation (DSC) algorithm. The rotational velocity of a rotating object is determined by two temporal-delay numbers at the minima of two DSCs that are derived from the four output signals of the quadrant photo-detector, and the sign of the calculated rotational velocity directly represents the rotational direction. The DSC algorithm does not require any multiplication operations. Experimental calculations were performed to confirm the proposed evaluation method. The calculated rotational velocity, including its amplitude and direction, showed good agreement with the given one, which had an amplitude error of ~0.3%, and had over 1100 times the efficiency of the traditional cross-correlation method in the case of data number *N* > 4800. The confirmations have shown that the remote evaluation of rotational velocity can be done without any circular division disk, and that it has much fewer error sources, making it simple, accurate and effective for remotely evaluating rotational velocity.

## 1. Introduction

Rotational parameters, which include angular displacement, velocity and acceleration, can describe the performance, operation states and kinetic characteristics of rotating systems [1,2], such as the vortices of fluid substances, gear transfer systems, rotational machines and fan blades, *etc*. The exact measurement of transient velocity is vitally important for accurately controlling and monitoring the movements of some bodies, and for acquiring fault information in machine diagnoses [3]. Thus the method of sensing rotational velocity can be used for the analyses, monitoring and control of mechanical systems, swirling gas- or fluid flow, *etc*.

For sensing rotational or angular velocity, there exist methods using gyroscopes, marking methods, interferometry, correlation methods, circular grating sensing and spatial filtering methods, *etc*. The gyroscope method using a Sagnac-effect-based integrated gyroscope with small footprint has high resolution, and can sense transient angular velocity [4,5,6]. However it is not suitable for remote measurement and super-low velocity, and can be affected by environmental factors [7,8]. The interferometry methods, including Fabry-Perot cavities and Mach-Zehnder interferometers, have complicated structures and high precision fabrication requirements [9,10,11]. In the correlation method, the traditional cross-correlation (TCC) is often used to process the output signals of two identical sensors in the measured region [12,13,14], which provides non-contact measurements with low cost for monitoring gas-solid and solid-liquid flows [15,16,17,18]. The image matching method, in which a high-speed camera captures a series of instantaneous images to calculate velocity, also belongs to the correlation methods [19,20,21,22]. Correlation methods are less applied for remotely sensing transient angular velocity, due to the numerous multiplication operations required. Marking methods with optical labels (plus quadrant photodiode) or Hall-effect sensors mainly sense average rotational velocities with lower resolution or direction ambiguity [23,24], which can hardly measure instantaneous rotational velocity. The methods based on circular gratings like optical gratings [25,26] and magnetic grids can be employed to sense the transient angular velocity of target objects with high resolution [27,28]. Their circular gratings must be set up as circular division disks to be coaxially mounted on the measured object, and their resolution and precision are dependant on the grating grooves. Optical gratings are easily destroyed by tiny dust particles and greasy dirt. In some cases such as those of high-speed rotating machines or when measuring gas- or fluid flows, *etc.*, circular benchmarks cannot be mounted on the measured systems die to limitations in their structures, or their performances are possibly deteriorated. In the spatial filtering (SF) method [29], a center coefficient is obtained from the central frequencies of the output signals of two differential spatial filters. Then the rotational velocity is calculated with the central frequencies and the center coefficient, and its direction is determined by the center coefficient and one linear displacement direction. Rotational velocity measurements using the SF method are complicated, since they need to compute all the center coefficients, the central frequencies and the displacement direction.

This paper will present a novel approach for remotely evaluating transient rotational velocities with a quadrant photo-detector (QPD) and a differential subtraction correlation (DSC) algorithm proposed by us, which does not require any circular indexing plates or benchmarks. The QPD outputs four random signals, from which the amplitude and direction of the rotational velocity are obtained simultaneously by using the DSC algorithm that only needs addition and subtraction operations without any multiplication or division, which should make it faster than other correlation methods. This paper is organized as follows: Section 2 shows the rotational velocity sensing system; Section 3 describes its evaluation principle in detail; Section 4 presents some experimental calculations to verify the proposed approach, and the final section gives our conclusions.

## 2. System for Sensing Rotational Velocity

**Figure 1:**
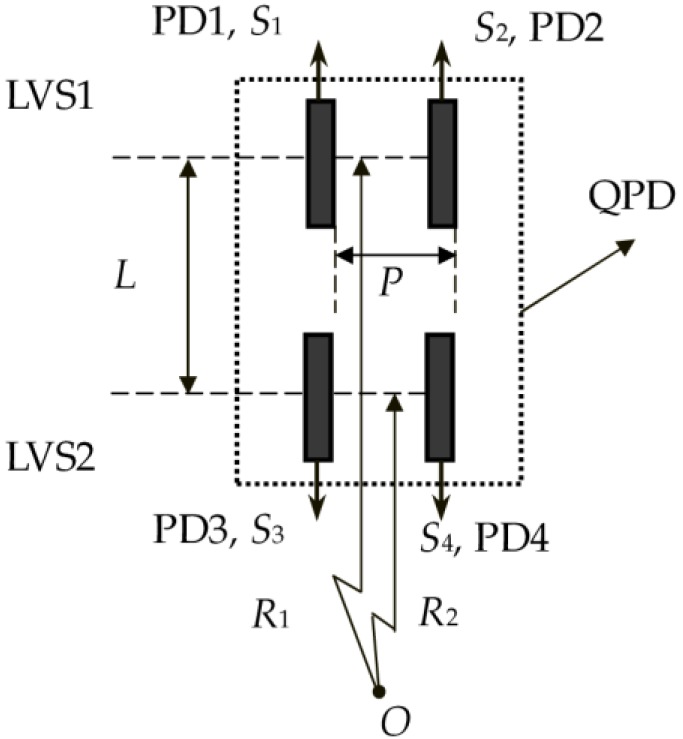
Structure of the QPD formed by four photovoltaic cells.

The quadrant photo-detector for sensing rotational- or angular-velocity, shown in Figure 1, is formed by four photovoltaic cells PD1, PD2, PD3 and PD4. PD1 and PD2 are arranged in parallel each other with distance *P*, which constitute a linear velocity sensor (LVS1), and output signals *S*_1_ and *S*_2_.

Similarly, PD3 and PD4 are also arranged in parallel each other with the distance *P*, which lead to another linear velocity sensor (LVS2), and output signals *S*_3_ and *S*_4_. The LVS1 and LVS2 are also aligned in parallel each other at the interval of *L*, and then form the QPD.

**Figure 2:**
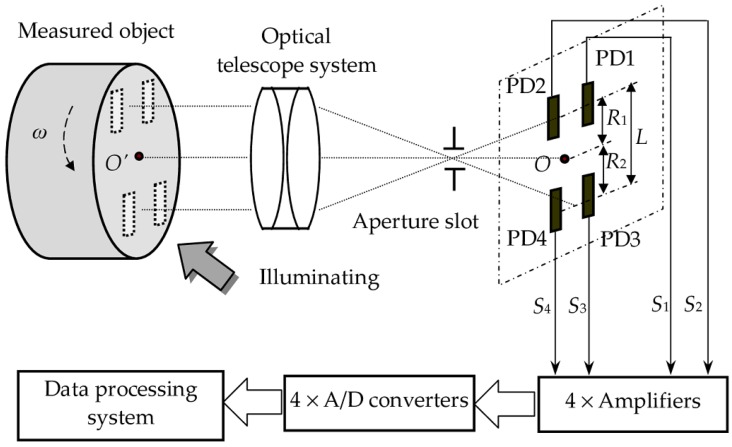
Schematic diagram of rotational velocity sensing system with DSC and the QPD.

The rotational velocity sensing system with the QPD and the DSC algorithm is schematically illustrated in Figure 2. Suppose the terminal plane of a measured object has a rough surface, and rotates around center *O’* with a rotational velocity ω. The surface of the terminal plane is imaged on the QPD through an optical telescope system with magnification *M*. The image of the measured object rotates around center *O* that is the imaged point of center *O’*, and it must cover the QPD. Thus the size of the terminal plane must be at least larger than *L*/*M*. The distances of center *O* to LVS1 and LVS2 are *R*_1_ and *R*_2_, respectively. The output signals of the QPD are random, owing to the stochastic reflection image of the terminal plane. The output signals *S*_1_, *S*_2_, *S*_3_ and *S*_4_ will go to the data processing system (DPS) after they are amplified and converted into digital signals. In the DPS, the DSC between *S*_1_ and *S*_2_ is calculated and noted as DSC1, which is related to the linear velocity of moving image on PD1 and PD2. The DSC between *S*_3_ and *S*_4_ is also obtained and denoted as DSC2, which indicates the linear velocity of moving image on PD3 and PD4. The rotational velocity ω of the measured object can be derived from the DSC1 and DSC2.

## 3. Principle of Evaluating Rotational Velocity with DSC

### 3.1. Image Velocity on QPD

**Figure 3:**
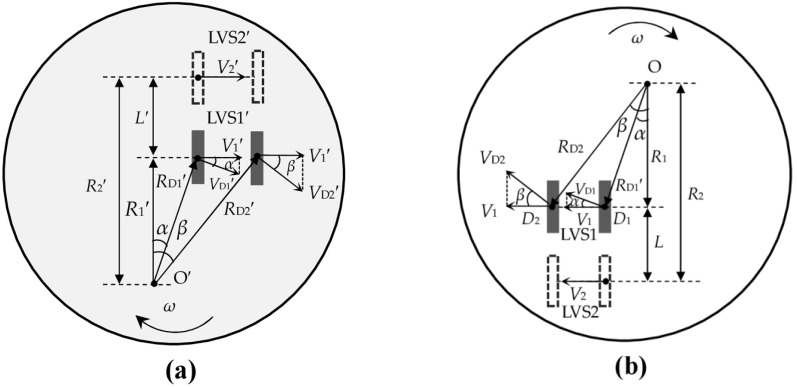
Relations of the rotational velocity ω with the linear velocities on (**a**) the terminal plane and (**b**) the image plane.

Supposing that the terminal plane of measured object rotates around the *O*’ in the rotational velocity of ω, the terminal plane and its imaged plane are depicted in Figure 3a,b, respectively, which are viewed from the optical telescope system. LVS1’, LVS2’ and *O*’ on the terminal plane are conjugated to LVS1, LVS2 and *O* on the image plane, respectively.

In Figure 3a, *D*’_1_ and *D*’_2_ are two arbitrary points on the central line of the LVS1’. The interval between *O’* and the central line of LVS1’ is *R*’_1_, and the distances of *O’* to *D*’_1_ and *D*’_2_ are *R*’_D1_ and *R*’_D2_, respectively. In Figure 3b, *D*_1_ and *D*_2_ are the conjugate points of *D*’_1_ and *D*’_2_, respectively, thus the distances of *O* to *D*_1_ and *D*_2_ are *R*_D1_ = *MR*’_D1_ and *R*_D2_ = *MR*’_D2_, respectively. With the rotational velocity of ω, the linear velocities at *D*_1_ and *D*_2_ are *V*_D1_ = ω*MR*’_D1_ = ω*R*_D1_ and *V*_D2_ = ω*MR*’_D2_ = ω*R*_D2_, respectively. *V*_D1_ and *V*_D2_ have the components *V*_D1_ × cosα and *V*_D2_ × cosβ, respectively, in the direction of LVS1, where α and β are the angles of *R*_1_ relative to lines *OD*_1_ and *OD*_2_, respectively.

Meanwhile, *R*_D1_ × cosα = *R*_D2_ × cosβ = *R*_1_, thus the image velocity *V*_1_ on any point of the central line of the LVS1 can be obtained, such that:

(1) $$V_{1}=\omega R_{1}$$

Similarly, we can also get the image velocity *V*_2_ on any point of the central line of the LVS2, governed by:

(2) $$V_{2}=\omega R_{2}$$

where *R*_2_ is the interval between *O* and the central line of LVS2. The image velocities *V*_1_ and *V*_2_ are insensitive to the variation of the amplification coefficient *M* which has been hidden in *R*_1_ and *R*_2_ on the image plane.

### 3.2. Characteristics of Output Signals of QPD

When a light source illuminates the terminal plane of the measured object that has a rough surface with finite and stochastic reflection, some light rays will be scattered by the rough surface, and then be imaged on the QPD through the optical telescope system with a high cut-off frequency in the spatial domain. The image on the QPD locating the image plane is randomly distributed, and has a wide spatial bandwidth. The output signal of each photovoltaic cell in the QPD is the integration of the randomly-reflected light intensity on its active area, which is also stochastic. Thus the QPD outputs four stochastic signals that are related to the random distribution of the rotating image, and the relations among the QPD output signals are involved with the image movement decided by the rotation of the terminal plane.

**Figure 4:**
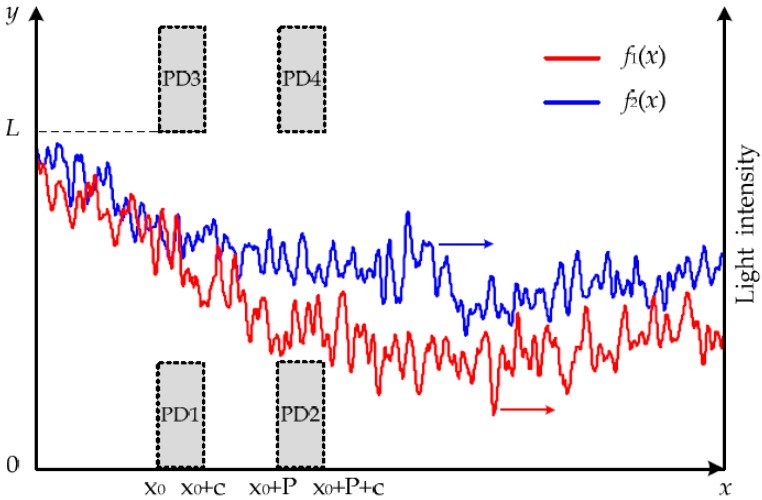
PD1–PD4 positions and the actual light-intensity distributions along LVS1 and LVS2.

It is assumed that the central lines of LVS1 and LVS2 are *x*-axes that are parallel with PD1–PD2 and PD3–PD4, respectively. Figure 4 shows schematically the stochastic light-intensity distributions on the active areas of the QPD whose PD1–PD4 positions are also indicated in *x*-*y* coordinate, where *f*_1_(*x*) and *f*_2_(*x*) are the examples of actual light-intensity distributions along the LVS1 and the LVS2, respectively, *x*_0_ is the *x*-axial position of PD1 and PD3, *c* is the width of the photovoltaic cells.

If only considering *x*-axial movement, the integration of the *y*-axially distributed random light at *x*-position can be taken as the whole light intensity at *x*-position, which ignores the *y*-axial factor and still is a stochastic distribution on the *x*-axis, shown as *f*_1_(*x*) or *f*_2_(*x*). When the rough surface of a measured body is imaged on the QPD, the QPD will be illuminated by the randomly distributed light that is regarded as only an *x*-axial distribution, and the output signals of the PD1–PD4 are the integrations of the light intensity distributions *f*_1_(*x*) or *f*_2_(*x*) in the *x*-axial widths of the PD1–PD4, respectively. If the measured body is rotating, its scattering light will be randomly changed, and then the QPD will output signals *S*_1_, *S*_2_, *S*_3_ and *S*_4_ that are stochastic in time domain.

If the LVS1 (PD1) is at position *x*_0_, its output signals *S*_1_ and *S*_2_ will be the integrations of *f*_1_(*x*) in the regions of PD1 and PD2, respectively, and they have the relation *S*_2_(*x*_0_) = *S*_1_(*x*_0_ − *P*). If the image on the LVS1 is moving in the image velocity *V*_1_, then the space-time relation *x*_0_ = *V*_1_*t* allows *S*_1_(*x*_0_) and *S*_2_(*x*_0_) to become time-domain signals *S*_1_(*t*) and *S*_2_(*t*), described by:

(3) $$\begin{array}{l} S_{1}(t)=\int_{x_{0}/V_{1}}^{(x_{0}+c)/V_{1}}f_{1}(V_{1}t)V_{1}dt\\ S_{2}(t)=\int_{(x_{0}+P)/V_{1}}^{(x_{0}+P+c)/V_{1}}f_{1}(V_{1}t)V_{1}dt \end{array}\}$$

**Figure 5:**
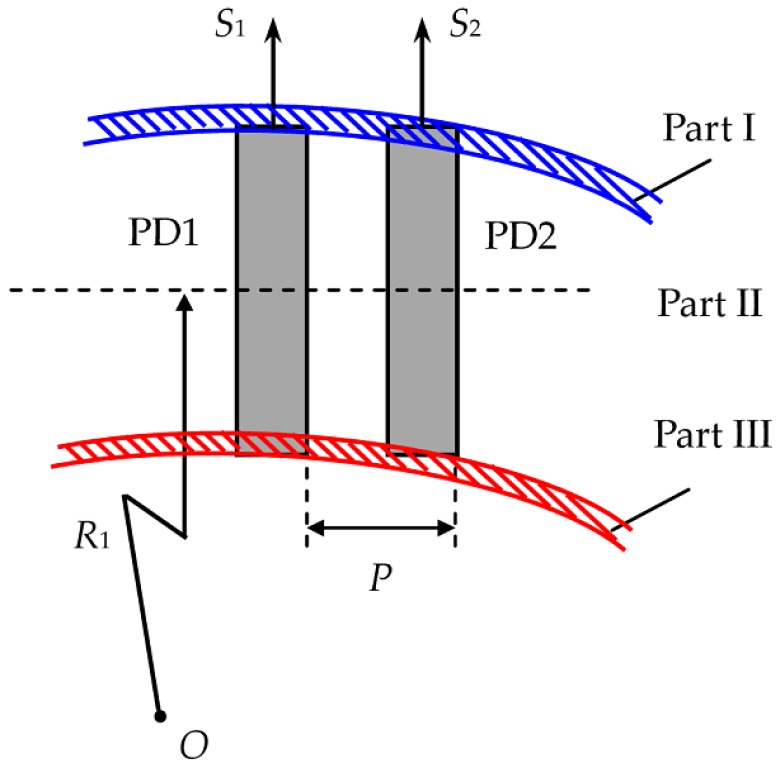
Region division of the rotational image on the LVS1.

The active-area factor of the photovoltaic cells cannot be ignored, thus the integrations in *S*_1_(*t*) and *S*_2_(*t*) will filter some high-frequency components of the light intensity distributions on PD1 and PD2, which actually function as low-pass filters that can remove much relevant characteristic information. When the measured object revolves around *O*’, its image on PD1 and PD2 can be divided into three parts: I, II and III, as illustrated in Figure 5.

Part I and Part III are labeled by blue slanted lines and red slanted lines, respectively, and Part II is the region between Part I and Part III. Part II with the largest area makes principal contribution to *S*_1_(*t*) and *S*_2_(*t*), and Parts I and III can be ignored because of their much less contributions. On other hand, the image of Part II on PD1 will be on PD2 after the delay time τ_1_ = *P*/*V*_1_. Thus Equation (3) indicates that *S*_2_(*t*) is approximately equal to the temporally delayed signal of *S*_1_(*t*) with the delay time of τ_1_, which can be written as:

(4) $$S_{2}(t)\approx S_{1}(t-\tau_{1})$$

where τ_1_ > 0 represents the movement direction from left to right, and τ_1_ < 0 represents that from right to left. Once τ_1_ is ascertained, the image velocity *V*_1_ on the LVS1 will be determined by:

(5) $$V_{1}=\omega R_{1}=P/\tau_{1}$$

Like the analogous *S*_1_(*t*) and *S*_2_(*t*), *S*_4_(*t*) is also approximately equal to the temporally delayed signal of *S*_3_(*t*) with the delay time of τ_2_, which is expressed to be *S*_4_(*t*) ≈ *S*_3_(*t* − τ_2_) where τ_2_ = *P*/*V*_2_ is the time delay of *S*_3_(*t*). τ_2_ > 0 also indicates the movement direction from left to right, and τ_2_ < 0 indicates that from right to left. Once τ_2_ is obtained, the image velocity *V*_2_ on the LVS2 will be also calculated with:

(6) $$V_{2}=\omega R_{2}=P/\tau_{2}$$

Based on the above descriptions, we can establish the features of the temporal output signals of the QPD: (1) they are random, corresponding to the spatially-stochastic reflection of the terminal plane; (2) the signals *S*_2_(*t*) and *S*_4_(*t*) are approximately interrelated to *S*_1_(*t*) and *S*_3_(*t*), respectively; (3) some high-frequency information consisting of more useful characteristics has been filtered, which is a disadvantage to correlation analysis. If the delay times τ_1_ and τ_2_ are obtained, Equations (5) and (6) may let the rotational velocity ω be calculated. According to the abovementioned features, the DSC algorithm is exploited to derive the delay times from the output signals.

### 3.3. Differential Subtraction Correlation

The output signals of the QPD are random and correlative, thus we also employ correlation analysis to calculate τ_1_ and τ_2_. However the conventional correlation method is low efficient, due to its multiplication operations with the complexity of about O(3*N*log_2_*N*) or O(*N*^2^), where *N* is the number of sampling data. In order to fast and exactly determine the delayed time, we propose the DSC to analyze the correlation ϕ_1_(τ) between *S*_1_(*t*) and *S*_2_(*t*), defined as:

(7) $$\varphi_{1}(\tau )=\int_{T_{1}}^{T_{2}}\left|\frac{dS_{1}(t-\tau )}{dt}-\frac{dS_{2}(t)}{dt}\right|dt=\int_{T1}^{T_{2}}\left|dS_{1}(t-\tau )-dS_{2}(t)\right|$$

where *T*_1_ and *T*_2_ are the start time and end time, respectively, of the integration. In practice, *S*_1_(*t*) and *S*_2_(*t*) are converted into digital signals *s*_1_(*n*) and *s*_2_(*n*), respectively, by using a multi-channel A/D converter with sampling interval *T*_0_. Thus the DSC ϕ_1_(τ) is discretized into Φ_1_(*m*) (*i.e.*, DSC1) as:

(8) $$\Phi_{1}(m)=\sum_{n=N_{1}}^{N_{2}-1}\left|s_{1}(n-m+1)-s_{1}(n-m)-s_{2}(n+1)+s_{2}(n)\right|$$

where *N*_1_ = *T*_1_/*T*_0_, *N*_2_ =*T*_2_/*T*_0_ and *m* = τ/*T*_0_ are integral. If *S*_2_(*t*) = *S*_1_(*t* − τ_1_), Φ_1_(*m*) will go to zero at *m* = *m*_1_ = τ_1_/*T*_0_, and is much more than 0 at other *m*, where *m*_1_ is an integer representing the temporal delay τ_1_.

Similarly, the output signals *S*_3_(*t*) and *S*_4_(*t*) of the LVS2 are converted into digital signals *s*_3_(*n*) and *s*_4_(*n*), respectively, with the same sampling interval *T*_0_. Then *s*_3_(*n*) and *s*_4_(*n*) are sent to the DPS to calculate the discrete DSC Φ_2_(*m*) (*i.e.*, DSC2) between *s*_3_(*n*) and *s*_4_(*n*), given by:

(9) $$\Phi_{2}(m)=\sum_{n=N_{1}}^{N_{2}-1}\left|s_{3}(n-m+1)-s_{3}(n-m)-s_{4}(n+1)+s_{4}(n)\right|$$

If *S*_4_(*t*) = *S*_3_(*t* − *τ*_2_), Φ_2_(*m*) will also go to zero at *m* = *m*_2_ = τ_2_/*T*_0_, and is much more than 0 at other *m*, where *m*_2_ is an integer representing the temporal delay τ_2_.

In practice, the output signals of the QPD in this rotational velocity sensing system have the relations *S*_2_(*t*) ≈ *S*_1_(*t* − *τ*_1_) and *S*_4_(*t*) ≈ *S*_3_(*t* − *τ*_2_), which let Φ_1_(*m*) and Φ_2_(*m*) go to the minimums at *m* = *m*_1_ and *m* = *m*_2_, respectively, and they are much more than 0 at other *m*. Based on Equations (8) and (9), the DSC algorithm is then described as follows:

(1) Acquiring *s*_1_(*n*), *s*_2_(*n*), *s*_3_(*n*) and *s*_4_(*n*) by discretizing the output signals of the QPD with synchronous four-channel A/D converter;

(2) Calculating the DSC1 and DSC2 at different *m*, according to Equations (8) and (9) with *s*_1_(*n*), *s*_2_(*n*), *s*_3_(*n*) and *s*_4_(*n*);

(3) Searching the minimums of Φ_1_(*m*) and Φ_2_(*m*), and taking the serial numbers at the minimums of Φ_1_(*m*) and Φ_2_(*m*) as the temporal-delay numbers *m*_1_ and *m*_2_, respectively.

The temporal-delay numbers *m*_1_ and *m*_2_ have indicated the delay times τ_1_ and τ_2_, respectively. The proposed DSC algorithm just requires subtraction, absolute and addition operations without any multiplications or divisions, so it can facilitate the fast correlation calculation of two random signals. Furthermore, its difference operation between two adjacent data can recover high-frequency components, and can eliminate low-frequency noise and shift, and then can enlarge the differences between adjacent data, which are advantageous to improve accuracy.

### 3.4. Evaluation of Rotational Velocity

According to the DSC algorithm described before, *m*_1_ and *m*_2_ can be derived from Φ_1_(*m*) and Φ_2_(*m*) by searching the minima of Φ_1_(*m*) and Φ_2_(*m*), respectively. Because τ_1_ = *m*_1_*T*_0_ and τ_2_ = *m*_2_*T*_0_, *m*_1_ > 0 and *m*_2_ > 0 indicate the image movement directions from left to right on the LVS1 and the LVS2, respectively, and *m*_1_ < 0 and *m*_2_ < 0 indicate that from right to left on the LVS1 and the LVS2, respectively. Thus Equations (1), (2), (5) and (6) lead to the relations of the rotational velocity ω with *m*_1_ and *m*_2_, governed by:

(10) $$\omega R_{1}=P/(m_{1}T_{0})$$

(11) $$\omega R_{2}=P/(m_{2}T_{0})$$

**Figure 6:**
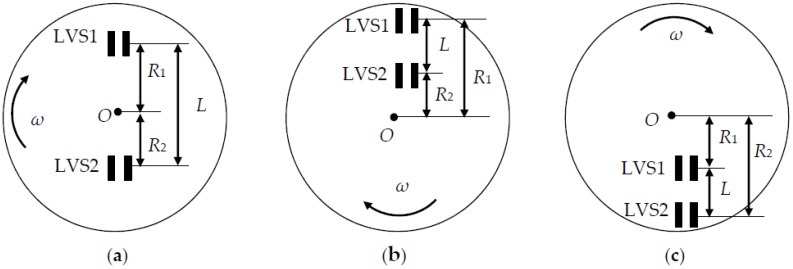
Locations of the rotational center *O* relative to the LVS1 and the LVS2: (**a**) *O* locates between LVS1 and LVS2; (**b**) *O* is below both LVS1 and LVS2; (**c**) *O* is above both LVS1 and LVS2.

Now let us suppose that the LVS1 is above the LVS2. The location of the rotational center *O* on the image plane may be between the LVS1 and the LVS2, below both the LVS1 and the LVS2, or above both the LVS1 and the LVS2, as shown in Figure 6a–c, respectively.

The signs of *m*_1_ and *m*_2_ represent the image movement directions on the LVS1 and LVS2, which can be used for judging the rotational center locations and directions. In Figure 6a, the sign of *m*_1_ is always different from that of *m*_2_, and *m*_1_ > 0 and *m*_1_ < 0 demonstrate the clockwise rotational direction and the counter-clockwise one, respectively. In Figure 6b,c, the sign of *m*_1_ is always the same as *m*_2_, and their rotational center locations can be discriminated by comparing *m*_1_ to *m*_2_, where |*m*_1_| < |*m*_2_| and |*m*_1_| > |*m*_2_| as indicated in Figure 6b,c, respectively, owing to the fact *R*_2_ ± *L* = *R*_1_. In Figure 6b, *m*_1_ > 0 and *m*_1_ < 0 illustrate the clockwise rotational direction and the counter-clockwise one, respectively. In Figure 6c, *m*_1_ < 0 and *m*_1_ > 0 mean the clockwise rotational direction and the counter-clockwise one, respectively. At the same time, Figure 6a–c have shown *R*_1_ + *R*_2_ = *L*, *R*_1_ − *R*_2_ = *L* with |*m*_1_| < |*m*_2_|, and *R*_2_ − *R*_1_ = *L* with |*m*_1_| > |*m*_2_, respectively, from which we can derive the rotational velocity ω as (1/*m*_1_ − 1/*m*_2_)*P*/(*LT*_0_) by using Equations (10) and (11). The sign of ω is the same as that of *m*_1_ in the cases of Figure 6a,b, and is opposite that of *m*_1_ in the case of Figure 6c, which shows that ω > 0 and ω < 0 always represent the clockwise rotational direction and the counter-clockwise one, respectively. Thus in all cases of different directions and centers, the sign of ω always indicates the rotational direction, and the rotational velocity ω can be determined by:

(12) $$\omega =\frac{P}{LT_{0}}\left(\frac{1}{m_{1}}-\frac{1}{m_{2}}\right)$$

Note that |*m*_1_| and |*m*_2_| are not equal to 0 in Equation (12), and must be less than *N* at least in the DSCs. In practice, they should be less than *N*/2, in order to accurately acquire them. Thus the better range of the rotational velocity calculated with Equation (12) should be from 2*P*/(*NT*_0_*R*_min_) to *P*/(*T*_0_*R*_max_), where *R*_min_ = min(*R*_1_, *R*_2_) and *R*_max_ = max(*R*_1_, *R*_2_).

The relations of ω with *m*_1_, *m*_2_, rotational center location and direction are listed in Table 1. The rotational velocity ω calculated with Equation (12) includes the amplitude and direction of the rotational velocity on the image plane, which is corresponding to the rotational velocity of the measured object. Therefore once *m*_1_ and *m*_2_ are obtained, the rotational velocity consisting of its amplitude and direction will be calculated by only using Equation (12).

## 4. Experimental Calculations

The rotation of a planar wooden disk with rough surface is taken as an example to confirm the validity of the proposed method for remotely sensing rotational velocity. The confirmation can be realized according to the following steps: (1) The wooden disk is installed on the shaft of a DC-micromotor (Model 2230G0003, Faulhaber GmbH & Co. KG, Schönaich, Germany) which drives the rotation of the disk. The angular speed of the DC-micromotor is controlled by the output voltage of an adjustable DC regulated power supply (Model APS3005S-3D, Atten Technology Co., Shenzhen, China); (2) The rotational velocity of the wooden disk is set by adjusting the output voltage and then measuring the rotational velocity with a DT-2234B Digital Tachometer (Suwei Co. Ltd., Guangzhou, China); (3) Sunlight or very bright white LEDs illuminate the wooden disk whose surface is imaged on a QPD through a telecentric imaging system (telescope) with magnification of 1×. The telecentric imaging system includes an adjustable aperture and two composite objectives with a focal length of ~26 mm. The QPD is constructed by four identical photovoltaic-cells with the active area of 0.89 × 4.39 mm^2^, where two photovoltaic cells of each linear velocity sensor (LVS1/LVS2) directly come from two adjacent pixels of a Si-based photovoltaic-cell array (Model A5V-38, OSI Optoelectronics Inc., Hawthorne, CA, USA), and then the LVS1 and LVS2 form the QPD. Other parameters of the QPD are as follows: *P* = 0.99 mm and *L* = 16 mm; (4) The output signals of the QPD are amplified and then converted into discrete data by four A/D converters with the sampling interval of *T*_0_ = 0.2 ms. The amplifier and the A/D converter are INA128 (Texas Instruments Inc., Dallas, TX, USA) and AD7663 (Analog Devices Inc., Norwood, MA, USA) with 16-bit resolution, respectively; (5) The discrete data are sent to a DPS, in which Φ_1_(*m*) and Φ_2_(*m*) are calculated, and their minimums are searched to get *m*_1_ and *m*_2_. Then the rotational velocity ω is calculated with Equation (12); (6) Finally, we compare the calculated value and direction to the given ones, and judge whether they are agree with each other.

**Figure 7:**
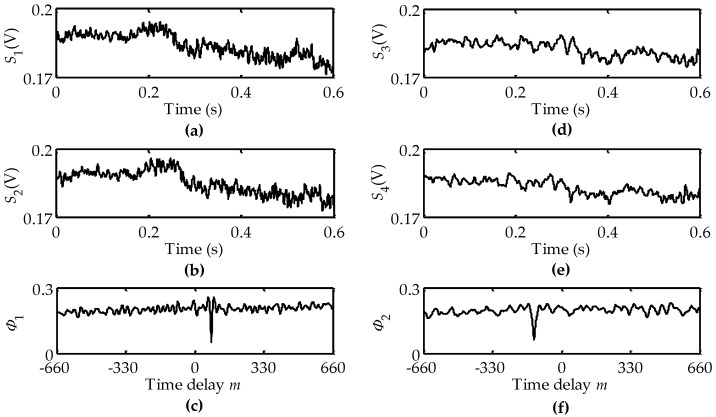
DSCs in the cases of clockwise rotation and the rotational center locating between the LVS1 and the LVS2: (**a**,**b**,**d**,**e**) are output signals of the QPD; (**c**) time delay between signals *S*_1_ and *S*_2_; (**f**) time delay between signals *S*_3_ and *S*_4_.

When the wooden disk was rotated at a rotational velocity of 6.3 rad/s in the clockwise direction, we located the rotational center *O*, whose distance to its closest LVS was ~6 mm, to be between the LVS1 and the LVS2. The output signals *S*_1_(*t*), *S*_2_(*t*), *S*_3_(*t*) and *S*_4_(*t*) of the QPD are shown in Figure 7a,b,d,e, respectively. Figure 7c,f illustrate the calculated Φ_1_(*m*) and Φ_2_(*m*), respectively. The minima of Φ_1_(*m*) and Φ_2_(*m*) were searched at *m*_1_ = 79 and *m*_2_ = −131, respectively. According to Equation (12), the calculated rotational velocity was 6.278 rad/s with the “+” sign that represents the clockwise direction. These results demonstrate that the calculated value and direction of the rotational velocity agree with the given ones.

**Figure 8:**
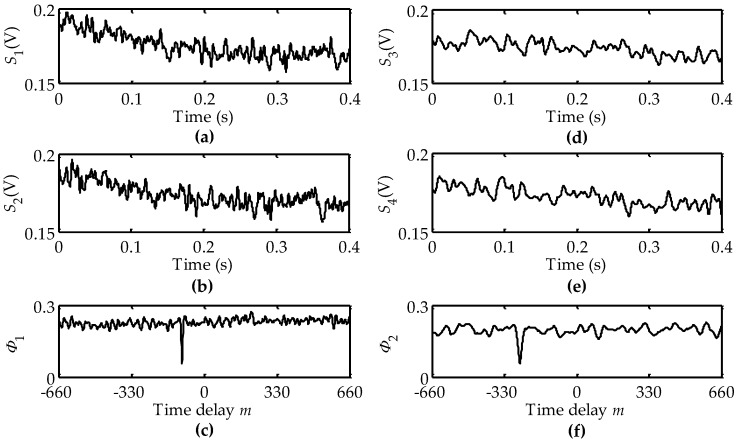
DSCs in the cases of the counter-clockwise rotation and the rotational center locating below both the LVS1 and the LVS2: (**a**,**b**,**d**,**e**) are output signals of the QPD; (**c**) time delay between signals *S*_1_ and *S*_2_; (**f**) time delay between signals *S*_3_ and *S*_4_.

When the wooden disk rotated counter-clockwise at a rotational velocity of 1.9 rad/s, we let the rotational center *O*, whose interval to its nearest LVS was ~10 mm, be below both the LVS1 and the LVS2. The output signals *S*_1_(*t*), *S*_2_(*t*), *S*_3_(*t*) and *S*_4_(*t*) are exhibited in Figure 8a,b,d,e, respectively. Figure 8c,f plot the calculated DSCs Φ_1_(*m*) and Φ_2_(*m*), respectively, from which we derived *m*_1_ = −100 and *m*_2_ = −260. Based on Equation (12), the calculated rotational velocity was −1.904 rad/s whose “−” sign indicates the counter-clockwise direction. The calculated rotational velocity including its amplitude and direction is also in agreement with the given one. Similarly, we performed the corresponding experimental calculations in the cases of other rotational directions and center locations, where the calculated rotational velocities were also in agreement with their given ones.

The abovementioned calculations had a relative amplitude error of ~0.3%. Their resolutions were up to 0.011 rad/s, and their optimal resolution was 5.4 × 10^−5^ rad/s (*N* = 4800). The resolution and its optimum of the DSC-based rotational velocity are actually determined by *P*/(*LT*_0_*m*_1_*m*_2_) and 4*P*/(*LT*_0_N^2^), respectively, which are related to the sampling interval *T*_0_ and the rotational velocity. For measuring super-low rotational velocities, the resolution can be improved to be excellent by increasing the sampling interval in the case of guaranteeing enough precision. In other often used methods, the resolution of laser gyroscopes can reach 0.01°/h~10°/h [4], and the TCC-based method has a relative error of 3.33% [12]. The marking method with an optical label plus quadrant photodiode possesses a relative error of 0.001% [24], while it measures only the average rotational velocity within the angular displacement of 2π rad. The circular-grating-based methods with Moiré optical grating and capacitive grating have relative errors of ~0.07% and 0.44%, respectively [30]. Compared to the existing method, the DSC-based method proposed has mid-ranking resolution and accuracy, whereas on the plus side, it does not require installing any standard division disk.

To verify the computational speed, the DSC-based and TCC-based calculations were performed in an Acer PC system equipped with an Intel dual-core i3-processer plus 3.4 GHz, OS Windows 10 and Matlab 2010. The DSC-based calculations needed average times of 141 ms and 86 ms in the cases of *N* = 4800 and *N* = 3600 data numbers, respectively, and the TCC-based ones consumed on average times of 158.92 s and 72.849 s, correspondingly. These achievements imply that the DSC-based computational speed has been improved up to 1127 times that of the TCC-based one in the case of a data number *N* > 4800. Thus the DSC-based calculation for rotational velocity is very fast and accurate, and is not affected by the movement and alignment error of the QPD relative to the measured object, making it fit for remotely evaluating transient angular-velocities. These features are due to the following reasons: (1) (1/*m*_1_ − 1/*m*_2_) always makes |*R*_1_ ± *R*_2_| equal to constant *L*, in the cases of arbitrary rotational center, eccentricity, radial fluctuation or parallel movement; (2) the amplification coefficient *M* has been hidden in *R*_1_ and *R*_2_ on the image plane, which leads to the insensitivity to various *M* changed by alignment error and axial shaking; (3) *V*_1_ ± *V*_2_ is always not related to eccentricity, parallel movement and radial shift. Thus the rotational velocity calculated with Equation (12) is only determined by pure rotation, which has much fewer error sources.

## 5. Conclusions

The method of remotely evaluating rotational velocity has been presented in this paper, where the rotational velocity is derived from the temporally-delayed serial-numbers at the minima of two DSCs that are calculated with the four stochastic output signals of the QPD formed by four identical photovoltaic-cells, and the sign of the rotational velocity represents the rotational direction. The DSC algorithm is implemented by differentiating and subtracting the random output signals of two photovoltaic cells, and then integrating the absolute value of the differential subtraction signal. In the DSC algorithm, the rough surface and random reflection of measured object will have a contribution to precisely determining the temporal delay and rotational velocity, owing to its difference operation. Experimental calculations were performed to confirm the proposed evaluation method. The calculated rotational velocities, including their amplitudes and directions, were in excellent agreement with their given ones, which possessed an amplitude error of ~0.3%, and had over 1100 times the TCC-based efficiency in the case of data number *N* > 4800.

The proposed evaluation method works without any circular indexing plate, and its DSC does not require any multiplication or division operations. At the same time, this method is insensitive to the radial fluctuation, axial shift, eccentricity and parallel movement of the measured object that has assembly or mismatch errors. The rotational velocity calculated with Equation (12) is only determined by pure rotation, which has many fewer error sources. Comparing to the SF method, this evaluation method does not require calculating central frequencies, center position and extra direction, and it can simultaneously determine the amplitude and direction of a measured rotational velocity. In general, the proposed method is simple, fast, accurate and effective for remotely evaluating transient rotational velocities.

## Figures and Tables

**Table 1:** The relations of ω with rotational direction, center location, *m*_1_ and *m*_2_.

Rotational Direction	Relation of *m*_1_ with *m*_2_	Judgment Results	
		Location of the Rotational Center *O*	Sign of ω
Clockwise	*m*_1_ > 0, *m*_2_ < 0	Between LVS1 and LVS2	+
	0 < *m*_1_ < *m*_2_	Below both LVS1 and LVS2	+
	*m*_1_ < *m*_2_ < 0	Above both LVS1 and LVS2	+
Counter-clockwise	*m*_1_ < 0, *m*_2_ > 0	Between LVS1 and LVS2	−
	0 > *m*_1_ > *m*_2_	Below both LVS1 and LVS2	−
	*m*_1_ > *m*_2_ > 0	Above both LVS1 and LVS2	−
